# Microcystins Exposure Associated with Blood Lipid Profiles and Dyslipidemia: A Cross-Sectional Study in Hunan Province, China

**DOI:** 10.3390/toxins15040293

**Published:** 2023-04-18

**Authors:** Shuidong Feng, Mengyue Cao, Peng Tang, Shuxiang Deng, Limou Chen, Yan Tang, Lemei Zhu, Xiang Chen, Zhijun Huang, Minxue Shen, Fei Yang

**Affiliations:** 1 Department of Epidemiology and Health Statistics, The Key Laboratory of Typical Environmental Pollution and Health Hazards of Hunan Province, School of Basic Medicine, School of Public Health, Hengyang Medical School, University of South China, Hengyang 421001, China; shuidong_f@hotmail.com (S.F.); mengyuec@126.com (M.C.); 2022000070@usc.edu.cn (P.T.); shuxiangdeng@163.com (S.D.); a2993287210@163.com (L.C.); jiayi1530@163.com (Y.T.); 2 School of Public Health, Changsha Medical University, Changsha 410219, China; zhulemei1228@163.com; 3 Department of Dermatology, Hunan Engineering Research Center of Skin Health and Disease, Hunan Key Laboratory of Skin Cancer and Psoriasis, Xiangya Clinical Research Center for Cancer Immunotherapy, Xiangya Hospital, Central South University, Changsha 410008, China; chenxiangck@csu.edu.cn; 4 Furong Laboratory, Changsha 410008, China; huangzj@csu.edu.cn; 5 Center of Clinical Pharmacology, The Third Xiangya Hospital, Central South University, Changsha 410013, China; 6 Hunan Provincial Key Laboratory of Clinical Epidemiology, Xiangya School of Public Health, Department of Social Medicine and Health Management, Central South University, Changsha 410000, China

**Keywords:** microcystins, triglyceride, cholesterol, lipoprotein, lipid profiles, dyslipidemia, cross-sectional study

## Abstract

Increasing evidence from experimental research suggests that exposure to microcystins (MCs) may induce lipid metabolism disorder. However, population-based epidemiological studies of the association between MCs exposure and the risk of dyslipidemia are lacking. Therefore, we conducted a population-based cross-sectional study involving 720 participants in Hunan Province, China, and evaluated the effects of MCs on blood lipids. After adjusting the lipid related metals, we used binary logistic regression and multiple linear regression models to examine the associations among serum MCs concentration, the risk of dyslipidemia and blood lipids (triglyceride (TG), total cholesterol (TC), high-density lipoprotein cholesterol (HDL-C) and low-density lipoprotein cholesterol (LDL-C)). Moreover, the additive model was used to explore the interaction effects on dyslipidemia between MCs and metals. Compared to the lowest quartile of MCs exposure, the risk of dyslipidemia [odds ratios (OR) = 2.27, 95% confidence interval (CI): 1.46, 3.53] and hyperTG (OR = 3.01, 95% CI: 1.79, 5.05) in the highest quartile was significantly increased, and showed dose–response relationships. MCs were positively associated with TG level (percent change, 9.43%; 95% CI: 3.53%, 15.67%) and negatively associated with HDL-C level (percent change, −3.53%; 95% CI: −5.70%, −2.10%). In addition, an additive antagonistic effect of MCs and Zn on dyslipidemia was also reported [relative excess risk due to interaction (RERI) = −1.81 (95% CI: −3.56, −0.05)], and the attributable proportion of the reduced risk of dyslipidemia due to the antagonism of these two exposures was 83% (95% CI: −1.66, −0.005). Our study first indicated that MCs exposure is an independent risk factor for dyslipidemia in a dose–response manner.

## 1. Introduction

The importance of managing dyslipidemia is gaining worldwide recognition due to its rapidly rising prevalence and disease burden [1,2,3]. From 1990 to 2017, the prevalence of dyslipidemia has been growing, and hyperbetalipoproteinemia has remained one of the leading causes of risk-attributable mortality worldwide [4]. Dyslipidemia is not only linked to total mortality, but it is also a risk factor for a wide range of diseases [5,6,7,8,9,10], including cardiovascular disease, stroke, diabetes, metabolic syndrome, pancreatitis, fatty liver, and others. The harm of dyslipidemia to health is mainly in the cardiovascular system. Cardiovascular disease is the main cause of mortality and disability globally [11]. Epidemiological data show that the coronary risk is anticipated to rise 2~3% for every 1 mg/dL (0.02 mM) drop in high-density lipoprotein cholesterol [12]. It can be seen that the harm of dyslipidemia to health is very significant. Diseases related to lipid metabolism, such as diabetes and dyslipidemia, are also on the rise, especially in developing nations [13]. Dyslipidemia prevention and management can greatly reduce disease morbidity and mortality [14,15,16].

There is now increasing attention being paid to the promoting effect of environmental exposure on dyslipidemia. Our previous studies have also found the influence of environmental factors (trace elements and toxic metals) on lipid markers [17]. In recent years, some epidemiological studies have found an intriguing phenomenon whereby serum triglyceride (TG) and total cholesterol (TC) levels exceed the normal range in certain fishermen who have consumed microcystin-contaminated lake water (Taihu Lake and Chaohu Lake) over a lifetime. This suggests that environmental factors (microcystins) may contribute to lipid damage [18,19]. However, analyses of the relationship between microcystins exposure and lipid levels or the risk of dyslipidemia in humans have not been reported. The corresponding epidemiological data are still lacking.

Cyanobacteria blooms and their secondary metabolites, microcystins (MCs), occur extensively and frequently around the world as a result of global warming and the eutrophication of water bodies; with more than 80 countries reporting cyanobacteria blooms [20,21,22], they are emerging as a serious public health hazard [23,24,25]. MCs were detected in 28 lakes and reservoirs in the ecological region of the eastern plain of China, including Chaohu Lake and Taihu Lake [26]. MCs are absorbed by plants through irrigation and eventually accumulate in the human body via the food chain [27]. More than 279 derivatives of MCs exist [28], and they slowly build up in the liver, intestines, kidney, heart, and immunological and reproductive systems, producing corresponding toxic effects that are harmful to both human and animal health [29,30,31,32,33,34]. Previous research has revealed that MCs pollution in Hunan Province endangers the health of the local inhabitants [35]. A few studies have observed hepatic steatosis and lipid metabolism disorders caused by MCs [36,37,38,39] and potential mechanisms [40,41,42,43]. Our earlier investigation revealed that chronic exposure to low amounts of MCs disrupted the hepatic lipid metabolism in mice [44].

Therefore, we performed a cross-sectional study in Hunan Province, China, to assess the effect of serum MCs levels on lipids and dyslipidemia.

## 2. Results

### 2.1. General Characteristics of the Study Population

The baseline characteristics of the 720 participants in this research are shown in Table 1. Among all the subjects, 35.4% had dyslipidemia, and the median of serum MCs concentration was 0.15 μg/L. The median of serum MCs concentration in the dyslipidemia group was 0.17 μg/L, which was significantly higher than that in the non-dyslipidemia group (0.15 μg/L, *p* = 0.002). The participants’ average age was 52.69 ± 14.93 years. Subjects with dyslipidemia were more likely to be older, have a higher education and income level, have a higher BMI and SSBs frequency, and have a higher prevalence of family history of dyslipidemia. Their mean plasma Zn and urinary Cd levels were higher, while their mean urinary Mo levels were lower compared with the subjects without dyslipidemia.

### 2.2. The Distribution of Lipid Profiles and the Prevalence of Dyslipidemia According to Serum MCs Concentration

According to the quartile of MCs concentration, participants were divided into four groups: Q1 (<25%), Q2 (25~50%), Q3 (50~75%), and Q4 (75~100%). MCs concentrations showed discrete distributions (as shown in Figure S1). Moreover, when the MCs concentration was in the quartile, it corresponded to multiple research objects at the same time (as shown in Table S1). For example, the MCs concentration of 22 subjects was equal to the 25th quartile (0.11 μg/L). Multiple research subjects whose MCs concentration values were in a certain quantile value were divided into the same group, resulting in slightly different numbers of people in each group. The lipid levels of participants with different serum MCs exposure levels are listed in Table 2. Compared with the Q1 group, TG levels in Q2, Q3, and Q4 groups were significantly higher, while HDL-C was significantly lower, with statistically significant differences (all *p* < 0.05). No statistical differences were observed in TC and LDL-C levels. Compared with the Q1 group, the prevalence of dyslipidemia, HyperTG, and HypoHDL-C in the Q2, Q3, and Q4 groups was also significantly increased (Table S2). These results suggest that MCs exposure may affect blood lipids.

### 2.3. The Associations of Serum MCs Quartiles with the Risk of Dyslipidemia

**Figure 1:**
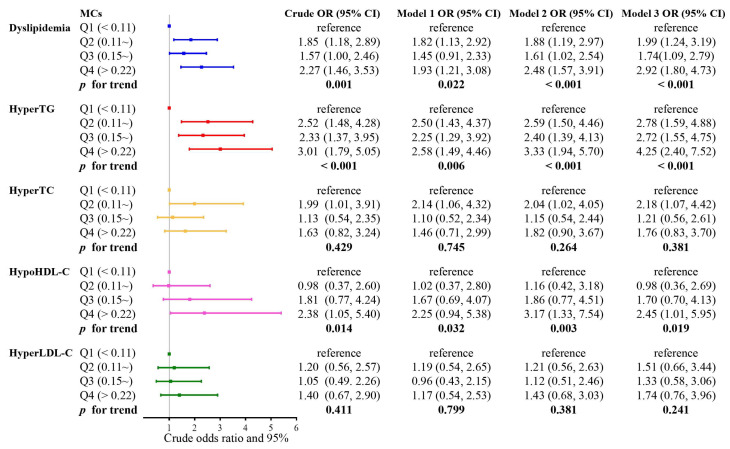
Odds ratios (ORs) with 95% confidence intervals (CIs) for the risk of dyslipidemia by quartiles of MCs concentration. Analysis of binary logistic regression with the exposures was categorized as follows: Q1 (<25%), Q2 (25~50%), Q3 (50~75%), and Q4 (75~100%). Model 1: adjusted for demographic variables including age, gender, educational level, annual family income, occupation, and BMI; Model 2: adjusted for variables related to lifestyle and disease history, including smoking, alcohol consumption, exercise, low intake of vegetables and fruits, SSBs frequency, family history of dyslipidemia, hypertension, history of chronic hepatitis, and diabetes; Model 3: adjusted for metals associated with lipids in our earlier study, including plasma Zn, plasma Se, plasma Fe, urinary Ti, urinary Mo, and urinary Cd.

**Figure 2:**
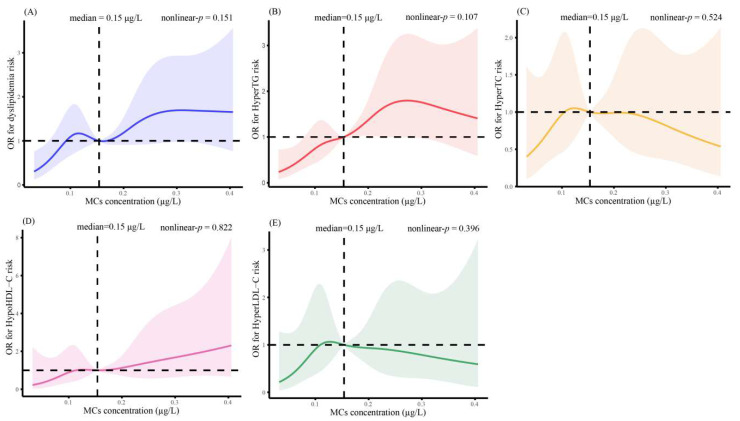
Restricted cubic spline plots: (**A**) The relationship between MCs and dyslipidemia risk; (**B**) The relationship between MCs and hyperTG risk; (**C**) The relationship between MCs and HyperTC risk; (**D**) The relationship between MCs and HypoHDL-C risk; (**E**) The association between MCs and HyperLDL-C risk. The colored solid line means the OR for dyslipidemia. Colored areas denote 95% CI. The ORs for dyslipidemia were calculated according to continuous MCs values, adjusted by Model 1, 2, and 3. Each MCs distribution has a knot at the 5th, 25th, 50th, 75th, and 95th percentiles.

The effects of MCs exposure on dyslipidemia are presented in Figure 1. The risk of dyslipidemia and hyperTG was 2.27 times (95% CI: 1.46, 3.53) and 3.01 times (95% CI: 1.79, 5.05) higher in the highest MCs quartile than in the lowest quartile, respectively. Consistent results were seen in both model 1 (OR = 1.93, 95% CI: 1.21, 3.08, and OR = 2.58, 95% CI: 1.49, 4.46), model 2 (OR = 2.48, 95% CI: 1.57, 3.91, and OR = 3.33, 95% CI: 1.94, 5.70), and model 3 (OR = 2.92, 95% CI: 1.80, 4.73, and OR = 4.25, 95% CI: 2.40, 7.52), which adjusted more comprehensively for a range of demographic, lifestyle, disease history, and metals confounders, respectively. The risk of dyslipidemia, hyperTG, and hypoHDL-C, as analyzed by the trend test, monotonically increased with increasing MCs quartiles across all models (*p* for trends < 0.05 for all models). The nonlinear *p*-value (nonlinear-*p*) was the threshold for the statistical significance of restricted cubic splines, if the nonlinear-*p* < 0.05 indicated that there was a non-linear relationship between MCs exposure and the risk of lipid abnormalities. In our results, all the nonlinear-*p* were > 0.05, indicating no non-linear relationships between MCs exposure and the risk of lipid abnormalities (Figure 2). Moreover, the *p* for the trend in Figure 1 also suggested a linear trend between MCs and the risk of dyslipidemia, high TG, and low HDL-C (*p* for trend < 0.05 for all models). Therefore, we believe that there is a linear dose–response relationship between serum MCs concentration and the risk of dyslipidemia, hyperTG, and hypoHDL-C.

### 2.4. Association of Serum MCs with Blood Lipids Levels

**Figure 3:**
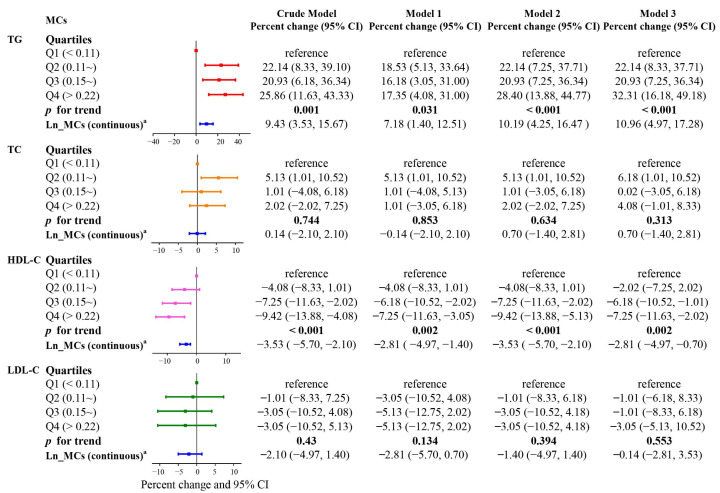
Association between serum MCs concentration and lipid profile. ^a^ Serum MCs concentrations were naturally logarithmically transformed to approximate normal distribution and then used as continuous variables in linear models to examine the relationship between MCs exposure (each doubling of serum MCs levels) and naturally ln-transformed blood lipids.

Figure 3 presents the estimated associations between blood lipid profiles and serum MCs levels using multiple linear regression analyses. Percent changes are the coefficient of the multiple regression linear model, which is the form of formula transformation. If the percent change > 0, it meant that MCs was positively correlated with blood lipid level; if the percent change < 0, it meant a negative correlation. The 95% CI can indicate whether the result is statistically significant. If it is not statistically significant, 95% CI of percent changes would include 1. As a continuous variable, serum MCs were significantly positively correlated with TG and negatively correlated with HDL-C in both crude and adjusted models. In the crude model, a doubling of the serum MCs level was associated with a 9.43% (95% CI: 3.53%, 15.67%) increase in TG and a 3.53% (95% CI: −5.70%, −2.10%) decrease in HDL-C, respectively. Similar patterns of associations were also discovered when the MCs level was analyzed as a categorical variable. Additionally, a monotonic increase or decrease was displayed in all models (*p* for trend < 0.05 for all models). Individuals whose serum MCs concentration was in the highest quartile showed a 25.86% (95% CI: 11.63%, 43.33%) rise in TG and a 9.42% (95% CI: −13.88%, −4.08%) drop in HDL-C compared to those in the lowest quartile. Categorical and continuous analyses found no correlation between TC and LDL-C levels and MCs.

### 2.5. Stratified Analysis

The stratified analysis revealed that MCs remained related to dyslipidemia risk. The strength of the connections varied somewhat across subgroups. Stronger connections were seen among individuals with demographic factors of age < 53 years, BMI ≥ 28.0, male, with an annual family income > CNY 30,000, and those exposed to smoking and drinking (Table 3). Furthermore, the risk of dyslipidemia increases monotonically with increasing quartile of MCs in the population aged less than 53 years, male, BMI less than 24 kg/m^2^, and never drinking (all *p* for trend < 0.05). After stratification by annual household income level and smoking status, the *p* for trend remained consistent with that before stratification.

### 2.6. Interaction of MCs with Metal Exposure

Our previous research found some metals related to blood lipids [17], and we compared the exposure levels of people with dyslipidemia to those without the condition (Table 1). In light of this, we also examined the combined effects of MCs and metals (plasma Zn, urinary Mo, and urinary Cd) on dyslipidemia risk (Table S3). The adjusted ORs for low MCs and high Zn levels, high MCs and low Zn levels, and for high MCs and high Zn levels, respectively, were 2.65 (95% CI: 1.56, 4.49), 2.33 (95% CI: 1.39, 3.91), and 2.18 (95% CI: 1.26, 3.77). The OR was calculated as an estimate of the relative risk of dyslipidemia; the relative excess risk of MCs plus Zn exposure was lower than the sum of MCs and Zn exposure alone [Relative excess risk due to interaction (RERI) = −1.81, 95% CI: −3.56, −0.05]. Based on our findings, there was an additive antagonistic effect for the combined effects of MCs with plasma Zn on dyslipidemia. Attributable proportion of interaction (AP) and 95% CI were −0.83 (−1.66, −0.005), which suggested that the 83% risk reduction in dyslipidemia risk may have been caused by the antagonistic effect of these two factors. There was no additive interaction effect between MCs and urinary Mo or MCs and urinary Cd on the risk of dyslipidemia.

## 3. Discussion

Our cross-sectional study indicated that MCs were an independent risk factor for dyslipidemia, showing dose–response relationships. Additionally, we discovered substantial associations between serum MCs concentration and blood lipid levels, with rising serum MCs concentration linked with increased TG and decreased HDL-C. As far as we know, epidemiological research on the association between MCs exposure and lipid levels or dyslipidemia are currently absent. This void is filled by the findings of our study. These results have important public health implications, as dyslipidemia has lately been recognized as an increased disease burden around the world, and they suggest that studies on the risk of dyslipidemia should focus not only on lifestyle and activity patterns but also on environmental factors.

Our research is from Hunan Province of China, a region with a hot and humid climate that promotes the existence of MCs in drinking water and food, thereby increasing human MCs exposure. For example, MCs pollution was found in Dongting Lake [45]. The median of MCs concentration in our study population’s serum was 0.15 μg/L. In comparison to populations living near Chongqing [46] and the Three Gorges Reservoir [47], the average serum MCs levels of our study population were lower, and higher than that of those living in Nanjing [30] and Hunan [48]. In all of these studies, the detection of MCs in serum was performed using an enzyme-linked immunosorbent assay (ELISA). Additionally, after validation, there was good consistency between MC detection by ELISA and HPLC-MS [49,50]. The discrepancies in MCs concentration among different studies or populations could be attributed to sample storage time, exposure length, dietary habits, and individual variance in microcystin toxicokinetics. Our study provides evidence that MCs can cause harm to human lipids even at low levels. According to recently published research, the prevalence of dyslipidemia was 28.2%, 29.8%, and 37.4% among the general population in Shengyang, Nanjing, and rural Henan Province [51,52,53]. In this research population, the prevalence of dyslipidemia was 35.4%, slightly higher than that of Chinese adults (34.0%) [54]. In addition, Hunan is the “hometown of nonferrous metals”, with the third biggest deposits of Pb-Zn ore in China [55]. Long-term mineral mining has led to the serious pollution of local surface water, soil and air [56]. In aquatic environments, MCs and Zn frequently coexist due to the continual discharge of industrial and agricultural residential waste [57]. Intriguingly, we observed a negative additive interaction between MCs and Zn exposure, indicating that the combined exposure to MCs and Zn might lower the risk of dyslipidemia. Previous studies have shown that MCs can induce lipid peroxidation [58,59,60], while Zn is an antioxidant and has a protective effect on lipid peroxidation [61,62,63]. Therefore, we speculated that the antagonistic effect of Zn and MCs on dyslipidemia may be due to zinc significantly alleviating the level of lipid peroxidation mediated by MCs. The specific mechanism needs to be further studied.

As of now, no direct epidemiological data exist to establish the relationship between MCs and the risk of dyslipidemia. In this population-based cross-sectional study, we found that MCs exposure is an independent risk factor for dyslipidemia, and that MCs were related to dyslipidemia risk in a dose–response manner. According to a survey conducted on fishermen by Chen et al. [18], 31.4% and 22.8% of fishermen, respectively, who drank MCs-contaminated lake water for their entire lives had abnormal TG and TC indexes. This suggests that MCs exposure may affect lipid levels. An earlier study revealed that TG and TC were more sensitive to MCs exposure than other markers of liver damage [19]. These research works align with the trend of our results. However, only about 35 people were included in their study and it did not explore the relationship between MCs exposure and lipid levels. According to our findings, MCs exposure was associated with higher TG levels and lower HDL-C levels, increasing the risk of dyslipidemia. Previous animal studies have confirmed that MCs can induce elevated plasma lipid levels in mice [19,42,64], affect liver and serum lipid metabolism, and lead to hepatic steatosis and lipid peroxidation [65,66]. Hepatic steatosis is a critical indication of improper lipid metabolism. These findings imply that environmental MCs pollution may increase the risk of dyslipidemia. Our study provided epidemiological data indicating that MCs pollution increases the risk of dyslipidemia.

However, the mechanism of how MCs influence the metabolism of lipids is finite and complex. Firstly, cross-omics techniques (metabolomics, transcriptomics, and metagenomics) discovered that the intragastric injection of MCs caused UFA production and PPAR activation-mediated hepatic lipid metabolism problems in mice [41]. Secondly, the endoplasmic reticulum (ER) is an important organelle that regulates fatty acid synthesis, cholesterol metabolism, and protein folding [67]. MCs exposure can induce endoplasmic reticulum stress in liver cells, which plays an important role in abnormal lipid metabolism in the mouse liver by altering the mRNA and protein expression of endoplasmic reticulum stress signaling molecules [42]. In addition, MCs can also induce NLRP3-mediated inflammatory responses in liver cells, which will increase the expression of IL-1*β* in cells and promote the progression of non-alcoholic steatohepatitis [68]. Other mechanisms include insulin resistance [38], intestinal microenvironment changes [66], and so on. Previous research by our team found that mRNA and protein expression levels related to lipid synthesis were increased in 6-week-old male C57BL/6 J mice exposed to various concentrations of MCs (0, 1, 30, 60, 90, and 120 μg/L in drinking water) for 9 months, while expression levels of fatty acids β-oxidation-related genes decreased after exposure to 60–120 μg/L MCs [44].

Our study, the first population-based study to establish the connection between MCs exposure and dyslipidemia, also provided epidemiological evidence of toxin-induced harm to blood lipids. However, some limitations to this study must be considered. To begin with, given the cross-sectional nature of this research, we cannot draw conclusions about a cause-and-effect relationship between MCs exposure and abnormal serum lipids. Second, there is the possibility of bias in the information gathered from the questionnaire, as well as in the sample selection process. Although it is difficult to exclude these biases, we did find a link between MCs pollution and dyslipidemia. Third, the enzyme-linked immunosorbent assay (ELISA) was unable to detect MCs derivatives [31], so we could not figure out which derivatives might be causing dyslipidemia. Larger, more fitting and multi-center prospective studies should be carried out to further verify the associations between MCs exposure and the risk of dyslipidemia and blood lipid profiles in the future.

## 4. Conclusions

This study first demonstrated that MCs exposure is an independent risk factor for dyslipidemia in humans. MCs exposure was associated with dyslipidemia risk in a dose–response manner. Furthermore, a linear relationship was found between serum MCs concentration and lipid levels. Our study provided a new environmental risk factor for dyslipidemia, which will contribute to the prevention and improvement of dyslipidemia, thereby reducing the occurrence and development of lipid metabolism-related diseases.

## 5. Material and Methods

### 5.1. Study Design and Population

Based on our previous census conducted in 2016–2017 in four regions of Hunan Province, China, we performed a population-based cross-sectional study. Details are available in published research reports [17,69]. Residents of the study area for at least five years, those aged >18, and those who completed questionnaires, blood draws for indicator tests, and serum MCs tests qualified as participants in our study. Patients with severe kidney or liver disease, those taking lipid- or glucose-lowering medications, and those who had recently had surgery were excluded. We initially screened 727 eligible adults. Subsequently, we further excluded those without complete information on the covariates of interest, such as the frequency of consumption of vegetables, fruits, beverages, etc. Finally, 720 participants were included in our analysis.

Written informed consent was obtained from all participants, and the research plan was green-lit by the Ethical Committee of Xiangya Hospital of Central South University (Institutional Review Board number: 2018081028).

### 5.2. Data Collection

A standardized questionnaire was employed to collect general characteristics and traditional risk factors by specially trained investigators who conducted in-person interviews with the participants. The information collected included, but was not limited to, the following: demographic data (e.g., gender, age, ethnicity, education level, occupation), lifestyle characteristics (e.g., smoking, alcohol consumption, fruit and vegetable intake or consumption, physical exercise), and history of diseases and family history (such as diabetes and hypertension). Simultaneously, health examinations were performed by qualified medical personnel to gather more potential health information, such as height, weight, and blood pressure. All participants had fasting blood drawn the morning of their regular physical examination, and their TG, TC, HDL-C, and LDL-C levels were analyzed in the clinical laboratory. Furthermore, about 5 mL of blood was collected in tubes without anticoagulant by trained professional nurses from nearby hospitals. All blood samples were centrifuged at 3000 rpm for 10 min to separate the serum from the blood clots, and then placed in individual Eppendorf tubes for long-term storage at −80 °C.

### 5.3. Serum MCs Concentration Detection

Based on prior authoritative works in the literature [46,47,70], serum MCs concentrations were determined using an enzyme-linked immunosorbent test (ELISA) kit [(#20-0068), Beacon Analytical Systems]. The serum MCs concentration testing procedure followed the kit instructions, as described in the study [35]. The kit had a detection range of 0.1~2 μg/L. The coefficient of variation for serum samples was 8.6%, and the average recovery was 99.6%.

### 5.4. Measurement of Lipid Markers and Diagnosis of Abnormal Blood Lipids

Using the biochemical automatic detector, the levels of TG, TC, HDL-C, and LDL-C were measured in accordance with the manufacturer’s instructions. The judgment of dyslipidemia referred to the standards of the “Guidelines for the Prevention and Treatment of Dyslipidemia in Adults in China (2016 Revised Edition)”: hyperTG was defined as TG ≥ 2.3 mM, hyperTC was defined as TC ≥ 6.2 mM, hypoHDL-C was defined as HDL-C < 1.0 mM, and hyperLDL-C was defined as LDL-C ≥ 4.1 mM. Dyslipidemia was defined as the presence of one or more of the above abnormal lipids.

### 5.5. Covariates and Models

We included demographic variables (age, gender, educational level, yearly family income, occupation, and body mass index) as covariates in model 1 based on prior research. Then, in model 2, we included the following lifestyle and illness history factors: smoking, alcohol use, exercise, a poor intake of vegetables and fruits, the frequency of sugar-sweetened beverages consumption, a family history of dyslipidemia, hypertension, a history of chronic hepatitis, and diabetes. Finally, model 3 adjusted for plasma zinc, plasma selenium, plasma iron, urinary titanium, urinary molybdenum, and urinary cadmium, which were related to blood lipids in our earlier study [17]. Body mass index (BMI) was determined by dividing weight in kilograms by the square of the height in meters (m). Smokers smoked daily and for over half a year, including current or former smokers. Alcohol drinkers drank alcohol at least once a week for over half a year. A low intake of vegetables and fruits was defined as less than 500 g per day [71]. The number of citations per week quantified sugar-sweetened beverages (SSBs) consumption. The disease history was defined by the physician’s diagnosis or self-reported use of related medication, or blood pressure 140/90 mmHg for twice measurement (hypertension), or fasting blood glucose 7.0 mM (diabetes). Any instance of the mother, father, or other relatives having dyslipidemia was considered as a family history of dyslipidemia.

### 5.6. Statistical Analysis

For a cross-sectional investigation, the sample size was estimated using the traditional statistical program PASS 15. The prevalence of dyslipidemia in our research population was approximately 35.4% (*p* = 0.35). We set the allowable error at 5% (the width of the confidence interval was 0.1, twice the allowable error) and set α to 0.05 [Confidence Level (1-Alpha = 0.95)]. The predicted number of samples was 366. The characteristics of all the subjects were described as a number with a percentage, the mean ± S.D (standard deviation) or median (IQR) (interquartile range). Comparisons between the individuals with or without dyslipidemia were assessed by the Chi-square test or Mann–Whitney U test based on the type of data. Given the skewed distributions of MCs concentrations and blood lipids (TG, TC, HDL-C, LDL-C), natural logarithm (ln) transformation was performed to approximate the normal distribution.

Binary logistic regression models were applied to evaluate the ORs and 95% CIs for dyslipidemia. The 95% CI has a similar effect to the *p* value, which can determine whether the results are statistically significant. If there was no statistical significance, the 95% CI of OR would include 1. For tabular aesthetics, we did not present the *p* values for Q2, Q3 and Q4 in Figure 1 and Figure 3. MCs concentrations were categorized based on the quartiles, with the lowest quartile as the reference group. Trend tests were performed to evaluate the linear dose−response by taking the median of each metal quartile as a continuous variable in the models. We further explored the dose–response relationship between MCs and dyslipidemia risk using restricted cubic splines with five knots at the 5th, 25th, 50th, 75th and 95th percentiles.

Multiple linear regressions were conducted to assess the relationship between MCs exposure levels and lipid profiles. Since both our dependent variables (TG, TC, HDL-C, LDL-C) and independent variable (MCs) were log-transformed, the results were re-transformed by exponentiating the coefficients and were presented as percent changes [72]. With MCs serving as a continuous exposure variable, using the formula [exp (ln2 × β) − 1] × 100% [73], the regression coefficients were converted and expressed as the percent change in blood lipids with each doubling of the serum MCs levels. On the other hand, the exposure variables were also divided into quartiles and introduced into the models as categorical variables for further analysis, and the percent changes were estimated by comparing each of the upper three quartiles to the lowest quartile using the formula [exp (β) − 1] × 100. The potential dose–response relationships between serum MCs levels and blood lipid levels were explored by trend analysis. The dummy variables were produced using the “Indicator” coding approach based on quartiles of raw serum MCs concentrations of the whole population, the lowest quartile of the MCs concentrations was selected as the reference group and coded as (0 0 0), the second quartile was coded as (1 0 0), the third quartile was coded as (0 1 0), and the highest quartile was coded as (0 0 1). In addition, subgroup analysis was performed by including the MCs and covariables in binary logistic regression models. Age (<53, ≥53), gender, annual family income (≤30,000 CNY, >30,000 CNY), BMI (<24, 24.0~27.9, ≥25), smoking (current or former, never) and alcohol drinking (current or former, never) were all included as stratification variables. Lastly, we analyzed the interaction effect by means of an additive model and calculated interaction-related indicators, including RERI and AP. RERI is the extra risk caused by interaction compared to the risk without interaction, RERI > 0 indicates that there is a positive additive interaction (that is, an additive synergistic effect), and RERI < 0 indicates that there is a negative additive interaction, namely an additive antagonistic effect. AP is the percentage of dyslipidemia cases that can be attributed to the interaction in people who are exposed to both factors. If there was no interaction, the 95% CI of RERI/AP would include 0 or RERI/AP = 0. If both AP and RERI were <0, a negative synergistic interaction existed. For statistical analysis, SPSS 25.0 (SPSS, Chicago, IL, USA) software and R (version 3.6.1) were used. MC concentrations in samples below the detection limits were imputed with a value equal to half the detection limit.

## Figures and Tables

**Table 1:** Comparison of prevalence of dyslipidemia according to different demographic characteristics of the study participants.

Characteristics	Total (n = 720)	Dyslipidemia		*p* Value ^a^
		Yes (n = 255)	No (n = 465)	
Age, years	52.69 ± 14.93	54.18 ± 15.40	49.98 ± 13.63	<0.001 ^b^
Gender, n (%)				0.266
male	501(69.6)	184 (72.2)	317 (68.2)	
female	219 (30.8)	71 (27.8)	148 (30.4)	
Ethnicity, n (%)				0.443
Han	582 (80.8)	210 (82.4)	372 (80.0)	
others	138 (19.2)	45 (17.6)	93 (20.0)	
Educational level, n (%)				0.039
primary school or below	271 (37.6)	78 (30.6)	193 (41.5)	
junior high school	161 (22.4)	63 (24.7)	98 (21.1)	
senior high school	191 (24.7)	76 (29.8)	115 (24.7)	
university degree or above	97 (13.5)	38 (14.9)	59 (12.7)	
Annual family income, CNY, n (%)				0.001
≤30,000	440 (61.1)	134 52.5)	306 (65.8)	
30,000–100,000	257 (35.7)	108 (42.4)	149 (32.0)	
>100,000	23 (3.2)	13 (5.1)	10 (2.2)	
Occupation, n (%)				0.510
physical labor	642 (89.2)	230 (90.2)	412 (88.6)	
mental labor	78 (10.8)	25 (9.8)	53 (11.4)	
Body Mass Index, kg/m^2^, n (%)				<0.001
<18.5	25 (3.5)	3 (1.2)	22 (4.7)	
18.5~23.9	299 (41.5)	66 (25.9)	233 (50.1)	
24.0~27.9	287 (39.9)	126 (49.4)	161 (34.6)	
≥28.0	109 (15.1)	60 (23.5)	49 (10.5)	
Smoking, n (%)				0.355
never	378 (52.5)	126 (49.4)	252 (54.2)	
former	68 (9.4)	23 (9.0)	45(9.7)	
current	274 (38.1)	106 (41.6)	168 (36.1)	
Alcohol drinking, n (%)				0.304
never	531 (73.8)	182 (71.4)	349 (75.1)	
former	47 (6.5)	15 (5.9)	32 (6.9)	
current	142 (19.7)	58 (22.7)	84 (18.1)	
Physical exercise, n (%)				0.371
yes	304 (42.2)	102 (40.0)	202 (43.4)	
no	416 (57.8)	153(60.0)	263 (56.6)	
Low intake of vegetables and fruits, n (%)	575(79.9)	206 (80.0)	369 (79.4)	0.647
SSBs Frequency (d/w), n (%)				0.015
<1	640 (88.9)	231 (90.6)	409 (88.0)	
1~2	61 (8.5)	17 (6.7)	44 (9.5)	
3~6	11 (1.5)	1 (0.4)	10 (2.2)	
>7	8 (1.1)	6 (2.4)	2 (0.4)	
Family history of dyslipidemia, n (%)				0.021
yes	18 (2.5)	11 (4.3)	7 (1.5)	
no	702 (97.5)	244 (95.7)	458 (98.5)	
Hypertension, n (%)				0.328
yes	196 (27.2)	75 (29.4)	122 (26.0)	
no	524 (72.8)	180 (70.6)	344 (74.0)	
History of chronic hepatitis, n (%)				0.277
yes	34 (4.7)	15 (5.9)	19 (4.1)	
no	686 (95.3)	240 (94.1)	446 (95.9)	
Diabetes, n (%)				0.741
yes	26 (3.6)	10 (3.9)	16 (3.4)	
no	694 (96.4)	245 (96.1)	449 (96.6)	
Serum MCs, μg/L	0.15 (0.11, 0.22)	0.17 (0.12, 0.24)	0.15 (0.11, 0.21)	0.002 ^b^
Dyslipidemia, n (%)	255 (35.4)	−	−	−
Plasma Zn, μg/L	891.24 (761.44, 1048.95)	928.13 (800.41, 1081.30)	866.91 (735.19, 1016.27)	0.001 ^b^
Plasma Se, μg/L	87.51 (67.05, 107.88)	90.51 (70.77, 106.88)	84.91 (62.31, 107.95)	0.119 ^b^
plasma Fe, μg/L	1052.63 (775.81, 1387.48)	1110.91 (872.72, 1373.08)	1023.71 (739.06, 1395.20)	0.051 ^b^
Urinary Ti, μg/L	137.28 (77.44, 193.13)	128.64 (71.62, 184.42)	142.40 (80.06, 201.88)	0.124 ^b^
Urinary Mo, μg/L	103.16 (59.00, 154.90)	84.94 (55.21, 165.37)	107.35 (61.72, 165.37)	0.020 ^b^
Urinary Cd, μg/L	4.72 (2.08, 8.44)	5.34 (2.00, 11.03)	4.58 (2.15, 7.37)	0.039 ^b^

Abbreviations: SSBs, sugar-sweetened beverages; Zn, zinc; Se, selenium; Fe, iron; Ti, titanium; Mo, molybdenum; Cd, cadmium. ^a^ From chi-squared test. ^b^ From Mann−Whitney U test. Data are presented as n (%) or mean ± SD/median (IQR).

**Table 2:** Characteristics of average levels of lipids in the study population by MCs quartiles (n = 720).

MCs (μg/L)	n	TG (mM)		TC (mM)		HDL-C (mM)		LDL-C (mM)	
		Mean ± SD	^a^ *p* Value	Mean ± SD	^a^ *p* Value	Mean ± SD	^a^ *p* Value	Mean ± SD	^a^ *p* Value
Q1	189	1.66 ± 1.23	ref	4.88 ± 0.89	ref	1.42 ± 0.33	ref	2.86 ± 0.83	ref
Q2	171	2.22 ± 2.85	0.003	5.54 ± 5.83	0.253	1.37 ± 0.28	0.130	2.90 ± 0.94	0.958
Q3	181	2.14 ± 2.10	0.002	4.92 ± 0.98	0.807	1.34 ± 0.30	0.002	2.79 ± 0.89	0.259
Q4	179	2.13 ± 1.54	<0.001	5.01 ± 0.94	0.259	1.30 ± 0.28	<0.001	2.83 ± 0.92	0.155

Abbreviations: TC, total cholesterol; TG, triglyceride; HDL-C, high-density lipoprotein cholesterol; LDL-C, low-density lipoprotein cholesterol; data are presented as mean ± SD. ^a^ *p* value from Mann−Whitney U test. Participants were divided into groups according to the quartile concentration of MCs: Q1 (<0.11 μg/L), Q2 (0.11 μg/L~), Q3 (0.15 μg/L~), and Q4 (>0.22 μg/L).

**Table 3:** Stratified analysis for the association between dyslipidemia and quartiles of MCs concentration.

Variables	Adjusted Odds Ratios (95% CI) by Quartile of Serum MCs Concentration (μg/L)				*p* for Trend
	Q1 (n = 189)	Q2 (n = 171)	Q3 (n = 181)	Q4 (n = 179)	
Age					
<53	reference	3.75 (1.84, 7.64) ***	3.41 (1.66, 7.00) **	4.12 (2.01, 8.44) ***	0.001
≥53	reference	1.23 (0.48, 3.12)	0.67 (0.26, 1.77)	2.31 (0.91, 5.90)	0.104
Gender					
male	reference	3.75 (1.96, 7.17) ***	2.36 (1.24, 4.48) **	3.30 (1.68, 6.48) **	0.009
female	reference	0.46 (0.15, 1.46)	0.56 (0.18, 1.69)	1.78 (0.60, 5.27)	0.109
Annual family income, CNY					
≤30,000	reference	1.48 (0.73, 2.99)	1.28 (0.64, 2.58)	2.14 (1.07, 4.29) *	0.043
>30,000	reference	3.86 (1.59, 9.37) **	1.99 (0.82, 4.81)	4.38 (1.70, 11.26) **	0.011
Body Mass Index, kg/m^2^					
<24	reference	2.43 (0.98, 6.03)	2.25 (0.87, 5.83)	3.01 (1.16, 7.83) *	0.043
24.0~27.9	reference	1.37 (0.63, 3.01)	0.78 (0.35, 1.72)	1.69 (0.75, 3.81)	0.297
≥28.0	reference	1.55 (0.08, 31.75)	11.29 (1.02, 124.77) *	7.13 (0.49, 104.30)	0.107
Smoking status					
current or former	reference	5.60 (2.43, 12.92) ***	2.28 (1.00, 5.18)	4.06 (1.67, 9.92) **	0.031
never	reference	1.09 (0.53, 2.25)	1.41 (0.70, 2.84)	2.55 (1.25, 5.18) *	0.005
Alcohol consumption					
current or former	reference	5.04 (1.45, 17.61) *	1.60 (0.50, 5.18)	3.65 (1.02, 13.06) *	0.181
never	reference	1.67 (0.91, 3.09)	1.61 (0.87, 2.99)	2.81 (1.52, 5.24) **	0.002

Participants were divided into groups with different MCs concentrations by quartiles: Q1 (<25%), Q2 (25~50%), Q3 (50~75%), Q4 (75~100%). Confounding factors were adjusted according to model 1, 2, and 3. * *p* < 0.05; ** *p* < 0.01; *** *p* < 0.001.

## Data Availability

The data that support the findings of this study are available from the corresponding author upon reasonable request.

## Supplementary Materials

The following supporting information can be downloaded at: https://www.mdpi.com/article/10.3390/toxins15040293/s1, Figure S1: Histogram of MCs concentration; Table S1: Frequency distribution of the MCs quartiles; Table S2: Distribution of dyslipidemia and its subtypes by quartile of serum MCs concentration (n = 720); Table S3: Additive Model of the Combined Effects of MCs with metals on dyslipidemia.
