# Supplementary material for: Microcystins Exposure Associated with Blood Lipid Profiles and Dyslipidemia: A Cross-Sectional Study in Hunan Province, China
Source: Toxins (Basel). 2023 Apr 18;15(4):293. doi: 10.3390/toxins15040293 (PMC10143012; doi:10.3390/toxins15040293)
Supplement: Supplementary file 1 [file toxins-15-00293-s001.zip › toxins-2269259-supplementary.pdf]

Supplemental Material

Microcystins Exposure Associated with Blood Lipid Profiles and Dyslipidemia: A Cross-Sectional Study in Hunan Province, China

Shuidong Feng [1,†], Mengyue Cao [1,†], Peng Tang [1], Shuxiang Deng [1], Limou Chen [1], Yan Tang [1], Lemei Zhu [2], Xiang Chen [3,4], Zhijun Huang [4,5], Minxue Shen [4,6,*] and Fei Yang [1,6,*]

1 Department of Epidemiology and Health Statistics, The Key Laboratory of Typical Environmental Pollution and Health Hazards of Hunan Province, School of Basic Medicine, School of Public Health, Hengyang Medical School, University of South China, Hengyang 421001, China; shuidong_f@hotmail.com (S.F.); mengyuec@126.com (M.C.); 2022000070@usc.edu.cn (P.T.); shuxiangdeng@163.com (S.D.); a2993287210@163.com (L.C.); jiayi1530@163.com (Y.T.)

2 School of Public Health, Changsha Medical University, Changsha 410219, China; zhulemei1228@163.com

3 Department of Dermatology, Hunan Engineering Research Center of Skin Health and Disease, Hunan Key Laboratory of Skin Cancer and Psoriasis, Xiangya Clinical Research Center for Cancer Immunotherapy, Xiangya Hospital, Central South University, Changsha 410008, China; chenxiangck@csu.edu.cn.

4 Furong Laboratory, Changsha 410008, China

5 Center of Clinical Pharmacology, The Third Xiangya Hospital, Central South University, Changsha 410013, China; huangzj@csu.edu.cn

6 Hunan Provincial Key Laboratory of Clinical Epidemiology, Xiangya School of Public Health, Department of Social Medicine and Health Management, Central South University, Changsha 410000, China

* Correspondence: shenmx1988@csu.edu.cn (M.S.); phfyang@usc.edu.cn (F.Y.)

† These authors contributed equally to this work.

Number of pages: 4

Number of figures: 1

Number of tables: 3

Contents **Page**

Contents	Page
**Figure S1. Histogram of MCs concentration**	3
**Table S1. Frequency distribution of the MCs quartiles**	3
**Table S2. Distribution of dyslipidemia and subtypes by quartile of serum MCs concentration**	3
**Table S3. Additive Model of the Combined Effects of MCs with metals on dyslipidemia**	3

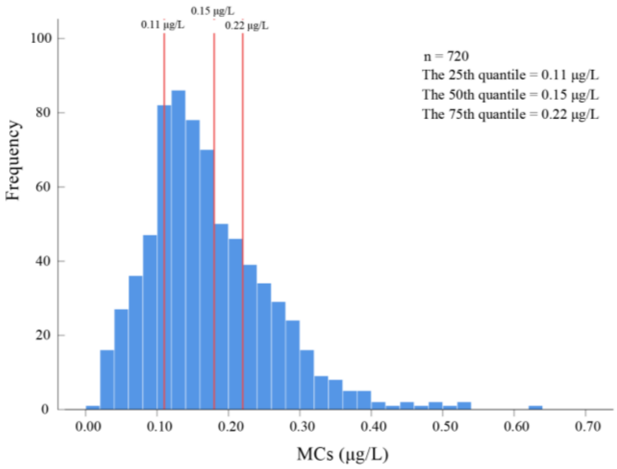

Figure S1. Histogram of MCs concentration

Table S1. Frequency distribution of the MCs quartiles

Percentiles	Concentration（μg/L)	Frequency (n)
The 25th percentile	0.110000	22
The 50th percentile	0.150000	20
The 75th percentile	0.220000	2

Table S2. Distribution of dyslipidemia and its subtypes by quartile of serum MCs concentration (n = 720)

MCs (μg/L)	n	Dyslipidemia		HyperTG		HyperTC		HypoHDL-C		HyperLDL-C	
		n (%)	[a] *p* value	n (%)	[a] *p* value	n (%)	[a] *p* value	n (%)	[a] *p* value	n (%)	[a] *p* value
Q1	189	48 (25.4)	ref	29 (15.1)	ref	16 (8.3)	ref	10 (5.2)	ref	14 (7.3)	ref
Q2	171	66 (38.6)	0.002	47 (28.3)	0.001	24 (14.5)	0.123	8 (4.8)	0.142	14 (8.4)	0.746
Q3	181	63 (34.8)	0.012	48 (26.8)	0.002	17 (9.5)	0.875	15 (8.4)	0.041	15 (8.4)	1.00
Q4	179	78 (43.6)	< 0.001	60 (33.7)	< 0.001	22 (12.4)	0.171	19 (10.7)	0.048	18 (10.1)	0.460

Data are presented as n (%). [a] *p* value from Chi-square test. Participants were divided into groups according to the quartile concentration of MCs: Q1 (< 0.11 μg/L), Q2 (0.11 μg/L ~), Q3 (0.15 μg/L ~), and Q4 (> 0.22 μg/L).

Table S3. Additive Model of the Combined Effects of MCs with metals on dyslipidemia

Variables	n (subjects)	AOR (95% CI)[b]	*p*	RERI (95% CI) [c]	AP (95% CI) [d]
MCs and Zn				−1.81 (−3.56, −0.05)	−0.83 (−1.66, −0.005)
low & low [a]	165	reference			
low & high	195	2.65 (1.56, 4.49)	< 0.001		
high & low	195	2.33 (1.39, 3.91)	0.001		
high & high	165	2.18 (1.26, 3.77)	0.005		
MCs and Mo				0.12 (−0.54, 0.77)	0.11 (−0.51, 0.73)
low & low	177	reference			
low & high	183	0.78 (0.48, 1.26)	0.31		

Table S3. *Cont.*

Variables	n (subjects)	AOR (95% CI)[b]	p	RERI (95% CI) [c]	AP (95% CI) [d]
high & low	183	1.16 (0.73, 1.84)	0.54		
high & high	177	1.05 (0.65, 1.70)	0.83		
MCs and Cd				0.33 (−0.34, 0.99)	0.26 (−0.26, 0.77)
low & low	179	reference			
low & high	181	0.88 (0.53, 1.45)	0.62		
high & low	181	1.07 (0.66, 1.73)	0.78		
high & high	179	1.28 (0.78, 2.09)	0.33		

Abbreviations: AOR, adjusted odds ratio; CI, confidence interval; RERI, relative excess risk due tointeraction; AP, attributable proportion; Zn, zinc; Mo, molybdenum; Cd, cadmium. [a] low & low was defined as those with MCs concentration lower than median (0.15 μg/L) and with metal concentration lower than median, and so on (the median of plasma Zn, urinary Mo and Cd were 891.56μg/L, 103.33 μg/L and 4.73 μg/L, respectively). Low MCs combined with low Zn exposures was considered as the unexposed reference category. [b] Adjusted by age, gender, educational level, annual family income, occupation, BMI, smoking, alcohol consumption, exercise, low intake of vegetables and fruits, SSBs frequency, family history of hyperlipidemia, hypertension, history of chronic hepatitis, diabetes. [c] RERI: the excess risk due to interaction relative to the risk without interaction. [d] AP: the attributable proportion of dyslipidemia due to interaction among individuals with both exposures. RERI ≠ 0, AP ≠ 0, indicate biological interaction.
